# Current Knowledge in Allergic Conjunctivitis

**DOI:** 10.4274/tjo.galenos.2020.11456

**Published:** 2021-02-25

**Authors:** Beatriz Vidal Villegas, Jose Manuel Benitez-del-Castillo

**Affiliations:** 1 Hospital Clinico San Carlos de Madrid Department of Ophthalmology, Madrid, Spain; 2 Universidad Complutense de Madrid, Instituto de Investigaciones Oftalmológicas Ramón Castroviejo, Madrid, Spain; 3 Clínica Rementería, Madrid, Spain

**Keywords:** Allergic conjunctivitis, allergic keratoconjunctivitis, contact blepharoconjunctivitis, vernal keratoconjunctivitis, atopic keratoconjunctivitis, ocular allergy

## Abstract

Allergic conjunctivitis is a disease of increasing prevalence that affects both children and adults and causes significant deterioration of their quality of life and sometimes irreversible visual damage. There are various forms of the disease, some are allergen-induced such as seasonal and perennial allergic conjunctivitis, giant papillary conjunctivitis, and contact allergic blepharoconjunctivitis, whereas others are not always explained by allergen exposure, such as vernal keratoconjunctivitis and atopic keratoconjunctivitis. We review their clinical course, characteristics, and differential diagnosis, and highlight recent advances in their pathophysiology and treatment.

## Allergic Conjunctivitis

Allergic conjunctivitis is a group of diseases caused by the ocular response to environmental allergens. They are common, affecting 10-20% of the population.^1,2^ Allergy rates are increasing and, at present, approximately 20% of the world population is affected by some form of allergy. Up to 40-60% of allergic patients have ocular symptomatology.^3^

Although allergic conjunctivitis usually does not affect vision, it causes important symptomatology and significantly reduce the quality of life of affected patients, especially children and adolescents because they are more commonly affected by some of the forms of the disease.^1^ Sometimes, however, severe forms can have a negative impact on vision if they develop a complicated course and affect the cornea, since it may result in corneal scarring and pannus. Hence, it is important that these diseases are diagnosed early and treated appropriately to improve patients’ quality of life, decrease the number of relapses, and avoid their possible complications.

Allergic conjunctivitis is usually bilateral with common eye symptoms and signs that include the following:^3^

- Itching, the hallmark of allergic eye disease

- Foreign body sensation

- Serous or mucous discharge

- Conjunctival hyperemia

- Tarsal papillary reaction

The symptoms can be differentiated into those that manifest primarily during the early or the late phase of the disease. Early signs are caused by coupling of histamine with its receptors and include: tearing, itching, redness, and edema (either conjunctival or palpebral), which are expressed by the acronym TIREd, first suggested by Fauquert.^4^ Late signs occur hours later and are characterized by epithelial infiltration with a variety of cells: lymphocytes, neutrophils, basophils and eosinophils. This later phase leads to chronic inflammation, manifested by photophobia, ocular pain, visual impairment, and discharge, which are expressed by the acronym POVD.^4,5^

Allergic conjunctivitis is the consequence of a type 1 allergic reaction.^5^ In sensitized individuals, when the allergen arrives at the conjunctiva it triggers the reaction: Th2-cells produce cytokines that induce immunoglobulin E (IgE) production by B-cells. The secreted IgE may bind to the membranes of mast cells and also to the allergen and provoke the secretion of inflammatory mediators.^5^

The classification of allergic conjunctivitis has been revised recently by the Ocular Allergy group of the European Academy of Allergy and Clinical Immunology (EAACI), which distinguishes two types of ocular surface hypersensitivity disorders: ocular allergy or ocular nonallergic hypersensitivity (Table 1).^6,7^ The first type, ocular allergy, can be caused by IgE-mediated or non-IgE-mediated mechanisms.^6,7^ IgE-mediated ocular allergy includes seasonal allergic conjunctivitis (SAC), perennial allergic conjunctivitis (PAC), vernal keratoconjunctivitis (VKC), and atopic keratoconjunctivitis (AKC). Non-IgE-mediated forms include contact blepharoconjunctivitis (CBC), VKC, and AKC. The second type, ocular non-allergic hypersensitivity, includes giant papillary conjunctivitis (GPC), irritative conjunctivitis, irritative blepharitis, and other borderline or mixed forms.

VKC and AKC are considered to be caused both by IgE-mediated and non IgE-mediated mechanisms. On the other hand, the different types of allergic conjunctivitis are sometimes related because patients that suffer from one form may later develop one of the other types of ocular hypersensitivity.

In the following sections, we will review the most common forms of allergic conjunctivitis, their clinical expression and management, and future prospects for their treatment (Table 2).

## Seasonal or Perennial Allergic Conjunctivitis

This is the most prevalent form of allergic conjunctivitis, with more than 95% of ocular allergy cases in the United States attributable to SAC and perennial acute conjunctivitis (PAC).^2,8,9^ Seasonal or perennial refer to the course of the disease, which is observed in both sexes and affects between 15% and 40% of the population.^9^ SAC, also known as hay fever conjunctivitis, is a bilateral acute disease usually due to outdoor allergens such as grass pollens and thus appears only in certain periods of the year that may vary with seasons and climate. PAC is also bilateral, but it is chronic, with exacerbation and remission periods, and is usually due to indoor airborne antigens, like dust mites or pet hair. The difference between the two conditions is simply the periodicity of symptoms; SAC is usually worse during spring through fall, abating in cold months, while PAC occurs throughout the year and is generally less severe.^2,5^ Both forms can also be mild, moderate, or severe depending on the intensity of symptoms and their impact on quality of life.^10,11^ However, more than half of patients report daily symptoms, and around 75% consider their symptoms to be severe.^12^

SAC and PAC are the ocular forms of a systemic allergic disorder (a type 1 IgE-dependent hypersensitivity) that it is usually manifested also in the respiratory system in the form of allergic rhinitis and/or asthma.^9^ Allergic rhinitis affects approximately 20% of the population and around 57% of patients with allergic rhinitis suffer from ocular symptoms, but allergic rhinitis is not a prerequisite for allergic conjunctivitis.^10,11,13,14^ However, allergic asthma, rhinitis, and conjunctivitis have a common pathophysiology, being the expression of an IgE-mediated allergy to airborne antigens. In sensitized individuals, allergen-specific IgE is bound to the surface of mast cells, so when the antigen binds to the receptors present in the membrane of these mast cells, they trigger the release of histamine and other preformed inflammatory mediators such as leukotrienes, prostaglandins, and other inflammatory mediators.^9^ The presence of specific IgE antibodies to airborne allergens can be demonstrated in almost all cases.^15^ An inflammatory response is then activated, and within 30 minutes there is an acute symptomatic reaction which is followed by a second delayed phase with recruitment of additional mast cells, eosinophils and other inflammatory cells to the conjunctiva that perpetuate the symptoms.^8^

**Figure 1:**
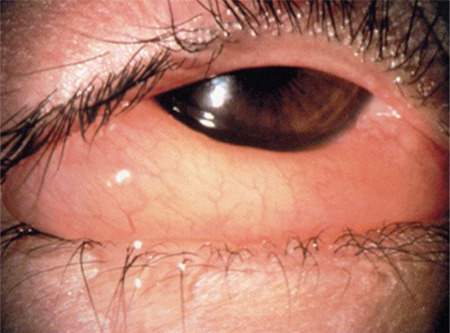
Chemosis in ocular hypersensitivity disorders

Patients experience periods of acute/subacute symptoms when in contact with the allergen, which resolve completely between attacks. TIREd (tearing, itching, redness, and edema) are the main symptoms, but there might be also photophobia, mild papillary reaction, chemosis (Figure 1), and palpebral edema.^4^ In this form of conjunctivitis, itching and chemosis are key symptoms, utterly disproportionate to the degree of hyperemia.^14,16^ Itching is typically worse in the nasal half of the conjunctiva, and the watery discharge may involve some mucus, which can be misleading.^17^ Corneal involvement is rare in SAC and PAC, although it may occur in severe forms.^6,18^

Ocular surface disorders such as dry eye disease, blepharitis, ocular rosacea, ocular toxicity from preservatives, or Meibomian gland dysfunction must always be included in the differential diagnosis of these diseases.^5,17,19^

The treatment must be directed towards allergen avoidance, symptom relief, and complication prevention. Anticipating allergen exposure such as seasonal spikes in pollen, may inhibit the inflammation before it becomes chronic and can generate sequelae such as dry eye or the development of AKC.^20^

## Vernal Keratoconjunctivitis

The VKC is a bilateral chronic inflammatory disorder that usually affects the upper tarsal or limbal conjunctiva. It is usually observed in tropical or mild/warm climates, but it can also be observed less frequently in cold climates.^16^ In Europe, there are between 1.2 and 10.6 cases per 10,000 individuals.^16^ It affects school-aged or prepubertal children, predominantly males, but in tropical regions may affect both sexes equally.^21,22^ It often evolves seasonally, with a maximum incidence at the end of spring and summer, suggesting a hypersensitivity reaction to pollen. However, there may be symptoms throughout the year, especially in warm climates where the condition can become perennial.^21,22^

VKC is caused by type 1 (IgE-dependent) and type 4 (IgE-independent) immune pathogenic mechanisms.^21,23,24^ Patients with VKC have an increased number of activated CD4+ T-lymphocytes, and, characteristically, of Th2, suggesting a type 4 hypersensitivity reaction.^22,23^ Children with VKC have been shown to have a higher prevalence of Ig deficiency and vitamin D deficiency; the latter could be explained by sun avoidance.^24,25^ Finally, 15-60% of affected children may also present with other atopic diseases.^21^

However, only 50% of the children affected have a sensitivity to aeroallergens. Thus, it is believed that this disease may have a complex etiology involving hyper-reactivity to allergens as well as various other environmental stimuli such as sunlight, wind, and dust.^8,14,16,21,23,24^ The ocular surface microbiome has also been implicated in the disease.^22^ Conjunctival scrapings show eosinophilic infiltration, but also mast cells, which are the predominant cell type in the substantia propria and are specifically increased in this type of conjunctivitis, inflammatory cytokines such as interleukin (IL)-6 and IL-8, and growth factors may have a role in the disease.^8,22,23,26,27^ Likewise, fibroblasts and epithelial cells are involved in tarsal papillae formation, while the limbal papillae may be due to inflammatory infiltrates.^22,28^

Although the pathogenesis of VKC is mostly immune-mediated, it is believed that an endocrine and/or genetic basis may also play a role in the disease, as demonstrated by the fact that it is more frequent in males, family-linked, and the limbal or palpebral forms depend on racial background.^23^

**Figure 2:**
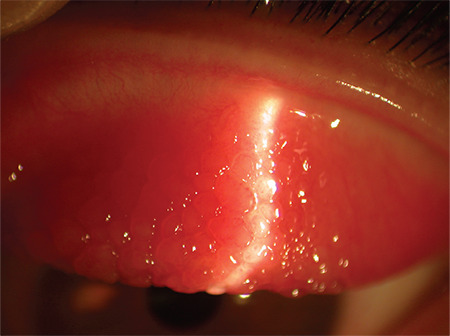
Tarsal papillae in vernal keratoconjunctivitis

VKC is classified clinically as tarsal, limbal, or mixed; the palpebral form is more frequent in Europe and the Americas, while the limbal type is the main form of presentation in African countries.^15^ Patients may have a personal history of allergies, asthma, atopic dermatitis, etc. In the tarsal form, giant papillae (&gt;1 mm) appear in the tarsal conjunctiva that can increase in size with time to become “cobblestone-like” papillae and are surrounded by mucus strings/goblets (Figure 2).^15,28^ In the limbal form, rounded nodules (also referred to as papillae) formed by lymphocytic infiltrates are observed in the limbus. At their vertex are collections of necrotic eosinophils, neutrophils, and mast cells that appear as white dots called Horner-Trantas dots.^16,21^ These dots normally appear when VKC is active and disappear when it abates between bouts of activity.^22^ Mixed forms show tarsal and limbal papillae. VKC may be complicated with punctate keratopathy that usually starts in the upper cornea and may evolve to form plaque or shield ulcers that can present as subepithelial white plaques. Since they are usually located in the superior or central cornea and thus affect vision, they may require surgery.^16,29^ Other possible sequelae of VKC are amblyopia, keratoconus, corneal scarring, and limbal stem cell deficiency.^29^

Typical findings in VKC include itching, redness, and watering or mucous discharge, like in all other forms of ocular allergy, but also photophobia and foreign body sensation, without involvement of the eyelid margins (Table 2), which is useful for differential diagnosis.^21,28^

Although VKC is a severe disease, it is also self-limited, subsiding around the age of 20 years. It also has an overall good prognosis, although up to 6% of patients will develop vision-threatening complications.^15,21,22^ The presence of giant tarsal papillae adversely affects the prognosis.^22,29^

## Atopic Conjunctivitis

**Figure 3:**
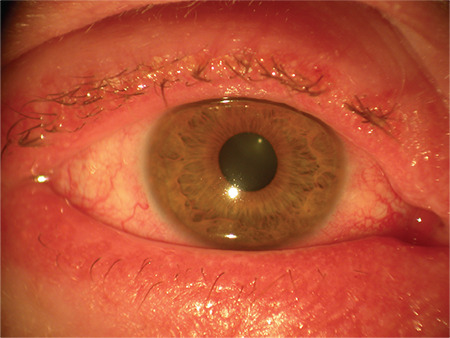
Atopic blepharokeratoconjunctivitis

AKC is the ocular manifestation of atopic dermatitis and is the most severe form of chronic allergic conjunctivitis.^1,8^ It is a bilateral inflammatory chronic keratoconjunctivitis and involves not only the ocular surface but also the eyelids, being therefore a blepharokeratoconjunctivitis (Figure 3). It is also a scarring disease with potential adverse ocular sequelae.^1^

AKC is more frequent in males and may occur at all ages, but there is a prevalence peak in patients between 20 and 50 years of age, with a personal or family history of atopic dermatitis or other allergic diseases such as eczema, asthma and/or urticaria.^1,28^ The percentage of patients with atopic dermatitis that develop AKC ranges from 25% to 42%.^1^

Patients with AKC usually show atopic dermatitis in the eyelids. Lid eczema results in lid hyperpigmentation (panda eyes), edema that causes horizontal lid creases (Dennie-Morgan lines), and absence of the lateral end of the eyebrows (Hertoghe’s sign).^1^ More advanced chronic disease may also cause keratinization of the eyelid margins, blepharitis, madarosis, tylosis, eyelid deformities, and reactive ptosis.^1^ The patients also show hyperemia, chemosis, and tarsal papillae, typically in the inferior tarsal conjunctiva and sometimes even Horner-Trantas dots in the limbus, especially in more acute phases.^1^ Conjunctival cicatrization can lead to symblepharon and shortening of the inferior conjunctival sac.^1^ The corneal involvement seems to be secondary to the conjunctival and palpebral involvement and may vary from superficial punctate keratitis to corneal ulcers, corneal scarring, and pannus.^1^

Patients complain of severe itching most of the year that is usually more severe during the winter months and in colder climates. There is also discharge that tends to be more aqueous than in VKC, but it may also be mucous.^1^

Chronic AKC leads to numerous complications: infections such as staphylococcal blepharoconjunctivitis and herpes simplex keratitis, cataracts (typically anterior subcapsular but also others), limbal stem-cell deficiency, keratoconus, glaucoma, retinal detachment, and corneoconjunctival tumors.^1^

AKC is a type 4 hypersensitivity immune reaction, with predominant participation of T-cells, and especially Th1-cells that produce chemotaxis and stimulate eosinophil production. Eosinophils initiate cytokine production, which heightens the inflammatory response.^1^ AKC is also considered to be due at least in part to an IgE-dependent mechanism.^6^ However, 45% of the patients with AKC do not display a hypersensitivity reaction to common allergens.^16^ AKC may result in dry eye that exacerbates itchiness and perpetuates conjunctival inflammation.^1^ Recent studies suggest that microbes and especially colonization of the conjunctiva with Staphylococcus aureus may have a role in the disease.^1^

## Contact Blepharoconjunctivitis

CBC is a severe blepharoconjunctival reaction caused by contact with an allergen. The patients develop acute inflammation of the palpebral skin and conjunctiva, hyperemia, burning, itching, and watery discharge in relation to a product applied topically, either over the eyelids or in the conjunctiva. The reaction may take several days to develop in the first exposure to the allergen. It is a type 4 delayed hypersensitivity reaction initiated by an exogenous allergen and mediated by Th1- and Th2-lymphocytes that secrete inflammatory cytokines.^16^ Identification of the allergen is most important, because treatment starts with avoidance/substitution of the allergen and anti-inflammatory therapy.

## Giant Papillary Conjunctivitis

GPC is a chronic inflammatory disease that courses with giant papillae on the upper tarsal conjunctiva. There is controversy over including GPC as part of ocular allergy surface disorders because GPC is sometimes caused by chronic mechanical stimulation of the conjunctiva and not by a hypersensitivity mechanism.^28^ For example, GPC has been observed in patients with ocular dermoids and filtering blebs and also in patients with inert substances in the ocular surface such as exposed sutures, scleral buckles, ocular prosthesis or contact lenses.^16,28^ Furthermore, the incidence of systemic allergy in GPC patients is comparable to that of the general population, and there is no increase in IgE or histamine in the tears.^16,28^ However, mast cells, eosinophils, and basophils are found in the conjunctiva of GPC patients, and they also show an increase of various immunoglobulins and cytokines, especially eotaxin in the tears.^29,30,31^ It is thus believed that the cause of GPC may be protein build-up on irregular surfaces and it is considered a nonallergic hypersensitivity disease.^6,16,30,31^

The symptoms of GPC include itching, foreign body sensation, watery or mucous discharge, mild conjunctival hyperemia, and development of a papillary reaction on the superior tarsal conjunctiva.^30^ There are various stages of the disease and usually there are no corneal complications, but superficial punctate keratitis or even shield ulcers and pseudoptosis may occur.^29,32^ Also, a personal history of atopy is a risk factor for GPC, so a detailed questioning and examination is important.^2^ Treatment is also slightly different for this subtype of conjunctivitis, because allergen avoidance plays a very important role.^29,31^

## Etiologic Diagnosis of Allergic Conjunctivitis

Because treatment of allergic conjunctivitis often depends on documentation of an allergy, it is important to investigate and identify which allergens the patient is allergic to. The first step is to do a thorough anamnesis in order to find out the allergens that cause the reaction. If the cause is clearly established, no more tests are needed.^6^ If further investigation is needed, even if no identifiable allergens have been found, the second step is skin prick or patch tests. Patch tests are preferred in CBC, while skin prick tests are used in the other diseases. These tests are carried out with a standard battery of allergens and sometimes with others that are not normally tested but suspected as the cause of the allergy. If skin testing is indicated but not recommended (e.g., the patient is taking antihistaminic systemic medications), or if results are ambiguous (e.g., presence of dermatographism), or simply to complement the results of previous SPT, serum specific IgE measurements for the aeroallergens can be considered.^5,6^

In case of doubt after systemic allergy evaluation tests, a conjunctival allergen provocation test (CAPT) may be of use to identify the etiology.^6^

In the CAPT, also known as conjunctival allergen challenge or ocular challenge test, an allergen is applied to the conjunctival mucosa to evaluate the patients’ immunoreactivity to the allergen. This test is used to confirm which allergens the patient is sensitive to and has the same scientific background as other provocation tests used extensively in other mucosae such as nasal or digestive.^6,33^ Non-specific or irritant challenges evaluate the hyperreactivity of the ocular mucosa, whilst direct mucosal challenges contain higher concentrations of the allergen encountered in environmental exposure and evaluate patients’ immunoreactivity to the allergen, following the guidelines for standard practice of the EAACI.^6,7,33^

A positive test will trigger the same signs and symptoms as those occurring when the allergen is encountered in real life, an IgE-mast cell-dependent immunoreactivity.^33,34^ CAPT is also useful to assess the relationship between symptoms and exposure in polysensitized patients and to assess response to therapy.^5^

## Treatment of Allergic Conjunctivitis

It is believed that although allergic conjunctivitis interferes with work, daily activities, and quality of life, a third of patients are not diagnosed and not treated.^5^ Because the prevalence of allergic diseases is rising, their impact on productivity and health costs is increasing and therefore there is increasing research and clinical trials on the subject.^5,9^ Although there are now very effective treatments for the acute forms of ocular allergy, the treatment of the perennial forms is still controversial. Recently, significant advances have been made in the treatment of severe or ocular allergy, particularly in immunomodulators and immunotherapy, which are the only disease-modifying treatments available and may provide lasting benefit.^5,7^

## Avoid Contact with Allergens

Nonpharmacologic treatments should always be the first approach and should accompany topical treatments as a first attempt.^17^ Complete allergen avoidance is the best bet, although it is frequently hard to enforce, and is especially important in PAC and SAC and also in VKC or in AKC when there is a documented allergy. It is also an issue in GPC, the signs and symptoms of which can be ameliorated by temporary discontinuation or shorter contact lens wearing periods, changing the contact lens cleaning solution, or refitting the patient with a different type of contact lens, especially daily disposable contact lenses.^29,31^ Punctal occlusion mechanical barrier gels may diminish the symptoms of allergic rhino-conjunctivitis and may help in treating non-specific factors that further worsen signs and symptoms, such as dry eye disease.^6,8^

## Non-pharmacologic Treatment

Cold compresses, saline, and cold artificial tears or ointments are useful because they alleviate the symptoms and dilute the allergen, especially in acute allergic conjunctivitis.^6,7,9^ Recent studies demonstrate the additive effect on the pharmacology of topical agents when combined with cold compresses and artificial tears.^7^ Other treatments such as ingestion of probiotics like mandarin orange yogurt or antagonists of the prostaglandin D2 receptor 2 have shown promising results in clinical trials, decreasing the symptoms of patients with rhino-conjunctivitis.^7,9^

## Topical Vasoconstrictors/Decongestants

The use of alpha-adrenergic agonists (especially those that bind to alpha-1 receptors) such as naphazoline, tetrahydrozoline, oxymetazoline, or brimonidine tartrate were amongst the first topical treatments to be approved for treatment of allergic symptomatology. They are sold over the counter and used to counteract hyperemia but are not recommended in adolescents and children (Table 3). They have a rapid onset of action and may be used in cases of episodic itching and redness but have a potential for inappropriate use by patients.^6^ They have a short duration and have many side effects such as tachyphylaxis, ocular irritation, and hypersensitivity.^6,7^ In our practice they are seldom indicated, should be used sparingly, and only as a short-term solution.

## Antihistamines, Mast Cell Stabilizers, and Dual Action Agents

### Antihistamines

There are in the market many antihistamine agents that can be topically administered but none has shown a clear advantage over the others, although clinical studies favor the use of dual action agents (antihistamine + mast cell stabilizers), especially if preservative free.^8,35^ Most frequently used are levocabastine, pheniramine maleate, and azelastine (Table 3).

Oral antihistamines such as loratadine, desloratadine, and fexofenadine are very effective in cases of allergic rhino-conjunctivitis. However, they have a higher frequency of systemic side effects such as sedation compared to topical antihistamines. They also cause a decrease in tear production, and thus may exacerbate the symptoms of conjunctivitis by inducing dry eye symptomatology.^6,7,35^

Some of the most potent antihistamines that are administered systemically such as cetirizine and bilastine have also been converted to ophthalmic preparations and are now in phase II-IV studies.^8^ Combinations of antihistamines with natural substances that have antioxidant and anti-inflammatory properties (such as catechin) and with substances that allow sustained release (such as cyclodextrin) have been suggested.^8^ Drug-loaded contact lenses have been produced for epinastine and olopatadine and may have a double action, both as allergen barrier and sustained-release delivery device, and thus have greater efficacy than eyedrops.^8^

Topical antihistamines, mast cell stabilizers, and dual action drugs are the first choice of treatment.^6,7^

## Mast Cell Stabilizers

Mast cell stabilizers inhibit mast cell degranulation and are therefore used as prophylaxis, with a loading period of around 2 weeks. The first drug of this type to be developed was cromolyn sodium, with subsequently developed drugs being more effective and having faster onset of action, such as nedocromil sodium, lodoxamide, pemirolast.^6,7^

## Dual Agents

Compared with antihistamines and mast cell stabilizers, topical dual-activity agents are clinically superior in both symptom relief and tolerability.^5^ They are considered first-line therapies and are the most commonly prescribed treatment.^5^

These agents block H1 receptors for acute therapy (antihistamine action) and inhibit mast cell degranulation for prophylaxis (mast cell stabilizer action). They include bepotastine, epinastine, azelastine, alcaftadine, and ketotifen, which are approved for itch treatment, and olopatadine, which is approved for all signs and symptoms of ocular hypersensitivity disorders.^6^ Olopatadine is safe, effective, and clinically superior to ketotifen, whilst some studies show that alcaftadine appears to be superior to olopatadine in reducing ocular itch.^7,8^

## Non-steroidal Anti-inflammatory Agents (NSAIDS)

NSAIDs can decrease symptomatology in allergic conjunctivitis but patients also report stinging/burning sensations when instilled and their use is therefore not widespread.^7,8^ Topical NSAIDs are generally used short-term, as an add-on to a topical antihistamine or dual-action agent.^6^

### Leukotriene Inhibitor

The leukotriene inhibitor montelukast when administered orally decreases the symptoms of PAC and SAC but is not as effective as oral antihistamines.^6,8,36^ Montelukast and oral aspirin have also been used in VKC (Table 4).^21,36^

## Steroids

Although they are the most effective anti-inflammatory agents in allergic conjunctivitis, they should be administered and monitored by an ophthalmologist only in severe or very acute forms of conjunctivitis and in short courses because of their frequent and severe ocular adverse effects (Table 4). There are many topical steroids, but the same drugs are not commercialized in different parts of the world. Prednisolone or dexamethasone are very efficacious, but it is preferable to use steroids with low strength and low effect on IOP such as fluorometholone, rimexolone, or loteprednol.^7,8^ There are two treatment regimens: pulsed or prolonged treatment. The pulsed therapy consists of 3-4 drops/day for 3-5 days and is frequently used for VKC and AKC.^37,38,39^ The prolonged therapy of 3-4 drops/day for 1-3 weeks and slow tapering may be used occasionally in severe chronic forms of disease.^6^ PAC and SAC rarely require the administration of topical steroids. Intranasal steroids are effective in reducing the nasal and ocular symptoms of SAC and PAC because the ocular symptoms may be due to a nasal-ocular reflex and there is little systemic absorption.^6,7,39^ Steroids applied to the skin may be used in AKC and CBC. Supratarsal injections of steroids in regular or depot formulations are effective in very recalcitrant cases AKC and VKC.^37,38^ Short courses of oral steroids are also effective in severe forms of AKC and VKC. However, supratarsal or oral steroids should be reserved for cases that do not respond to the other forms of therapy.^6,39^

There are also relatively new molecules, known as selective glucocorticoid receptor agonists or modulators, experimental drugs that share only the immunosuppressive and anti-inflammatory properties, with fewer side effects such as atrophy.^40^ There are various ongoing clinical trials for these new molecules and their applications, and some of these molecules have been found to suppress inflammation and allergic conjunctivitis in animal models.^40^

## Immunomodulators

The topical calcineurin inhibitors cyclosporine A and tacrolimus are very effective in the treatment of GPC, VKC, and AKC and may serve as steroid-sparing agents when these forms of chronic allergic conjunctivitis become steroid-dependent (Table 4).^1,6^

Cyclosporine A (CsA) is also used worldwide for the treatment of dry eye. Suspensions of CsA may be prepared in pharmacies, although some countries also have commercialized forms.^41^ The concentrations of CsA in the different ophthalmic formulations range between 0.01% and 2% and therefore the administration varies between 1 and 6 times per day.^7^ Tacrolimus may also be prepared as suspension by pharmacies and exists as an ointment for dermatological purposes in most countries at a concentration of 0.03-0.1%. Recent research shows that tacrolimus may have similar if not superior effectivity than CsA for the treatment of VKC. Moreover, dermatologic ointment containing tacrolimus is effective for the treatment of lid eczema in AKC.^1,6^ Topical treatment with calcineurin inhibitors has side effects such as stinging/burning sensation and the possibility of molluscum contagiosum virus, papillomavirus, or herpesvirus infection, although there is evidence from studies on dry eye syndrome that treatment with CsA can be topically administered long term and without systemic absorption.^6,7,8,41^ At present, tacrolimus is generally administered topically in cases that do not respond to CsA.^1^ Finally, in very severe cases, allergic conjunctivitis such as VKC and AKC may require systemic immunosuppression that is usually achieved with CsA, tacrolimus, or mycophenolate mofetil (Table 4).^1,6,39^

## Immunotherapy

The goal of immunotherapy is to diminish the symptoms and signs of rhinitis and conjunctivitis triggered by known allergens and to prevent their recurrence. Allergen-specific immunotherapy may be considered in cases of failure of first line treatments, or as a modifier of the natural course of the disease.^1,42,43^ Changes involve downregulation of Th2 response and upregulation of regulatory T-cells.^5^ It is carried out by administering gradually increasing amounts of the allergen to induce an immunological tolerance. According to the EAACI guidelines, it is indicated in patients with a documented IgE-mediated hypersensitivity to airborne agents, with severe forms of rhinoconjunctivitis that affect their quality of life in spite of allergen avoidance and pharmacotherapy.^11,32,44^ It can also be applied in children, but because it requires a strict regimen of desensitization, it may be difficult to treat children below 6 years of age.^17,43^ There are commercial forms of many recognized allergens and the allergist determines the allergen to be prescribed based on previous hypersensitivity tests. Desensitization consists of two phases, an induction phase that lasts 5-8 months and a maintenance phase that last 3-5 years.^43,44^ The standard method of administering the antigen has been subcutaneous injection (SCIT), but recently other less invasive methods have been developed, such as sublingual (SLIT) or epicutaneous administration, with good results.^8,43^ Adherence to SLIT is deemed better because it does not involve injections but has not been studied as exhaustively as SCIT; more randomized controlled trials are needed. Other forms of immunotherapy such as intralymphatic administration or edible vaccines are still being studied.^8,44^

In isolated allergic conjunctivitis (IgE- and non-IgE-mediated), allergen immunotherapy may be considered on the same premise as in rhinoconjunctivitis. However, there is less evidence of its beneficial effects and a few studies have documented an improvement of the clinical symptoms in VKC but not in AKC.^8,22,39,44^

## Biologicals

In theory, biological treatments could have superior results because they block the underlying inflammation pathways by bonding with specific biological molecules, whereas the above-mentioned treatments use unspecific ways of decreasing conjunctival inflammation.^40^ A few trials have reported the systemic use of the biologicals omalizumab, indicated for severe asthma, and dupilumab, indicated for atopic dermatitis, in VKC and AKC. Omalizumab shows generally good results, though it has not yet been approved for allergic conjunctivitis, while dupilumab may increase the risk of blepharoconjunctivitis, which is tacrolimus-responsive in patients with severe atopic disease or previous AKC.^1,36,45,46^ Benralizumab, mepolizumab, and reslizumab, which are anti IL-5 biologic agents have not been studied in the context of allergic conjunctivitis.^5^

Insunakinra (EBI-005) is the first inmunophilin synthesized for topical ophthalmologic use. It is an antagonist of the IL-1 receptor and binds to it, blocking the rest of the pathway. It has been documented to diminish ocular surface symptoms such as itching, inflammation, and discomfort.^47^

Another molecule called liftitegrast (Shire Pharmaceuticals) has both activity as an antagonist of the IL-1 receptor and as antagonist of the lymphocyte functional antigen-1 and has proven effective for treatment of ocular surface symptoms.^48^

## Surgery

In very recalcitrant cases of VKC and AKC, eye surgery may be needed. Papillae resection, in some cases with grafting of autologous conjunctiva, amniotic membrane or mucous membrane are effective in the treatment of severe forms of VKC with corneal ulcers.^32,49,50^ Plaque resection may be necessary for subepithelial deposits in VKC.^16,30^ In AKC, surgery may be needed for eyelid and conjunctival scarring.

Atopic disease and AKC can be complicated by subcapsular cataracts and/or severe ocular surface disease that may require complex surgery such as superficial keratectomy, limbal transplantation, or keratoprosthesis implantation.^51^

## Conclusions

To conclude, we would like to stress that in recent years there have been important advances in the knowledge and treatment of ocular allergy that allow the effective and safe management of most forms. Dual activity agents are considered first-line therapy; when symptoms are uncontrolled, doctors may consider a short course of topical steroids. Other treatments such as oral antihistamines or topical ophthalmic NSAIDs can be used alongside, and topical calcineurin inhibitors are used off-label as a next step. Immunotherapy can provide a long-term solution to the symptoms and should be considered when medical therapy is insufficient or ill-tolerated. However, there are unmet needs in the field, such as the standardization of the optimal doses of the treatments. Future pharmacologic developments are also expected, especially in immunomodulation and immunotherapy.

## Figures and Tables

**Table 1:**
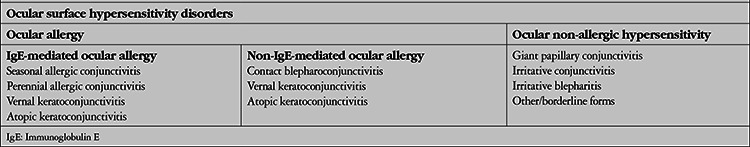
Classification of ocular surface hypersensitivity disorders

**Table 2:**
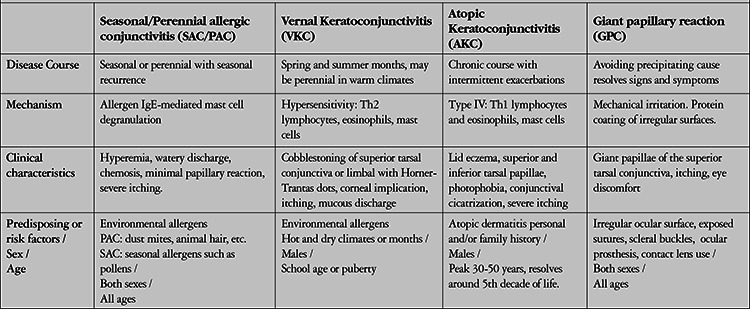
Characteristics of the different types of allergic conjunctivitis (adapted from Patel et al 2018). Abbreviations within the table.

**Table 3:**
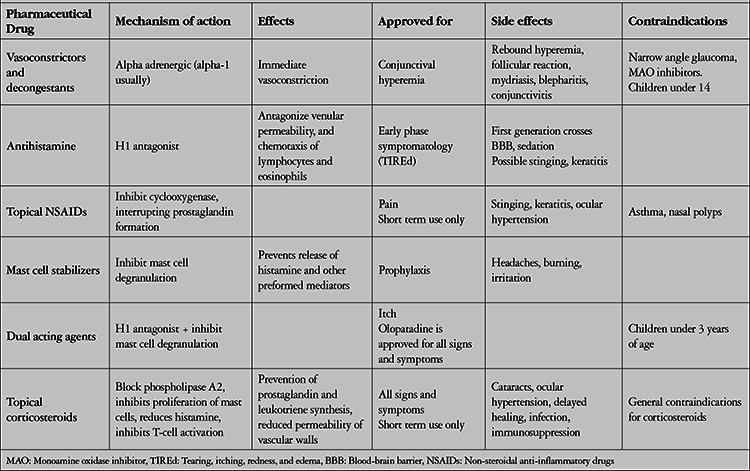
Topical pharmaceutical drugs currently in use for allergic conjunctivitis

**Table 4:**
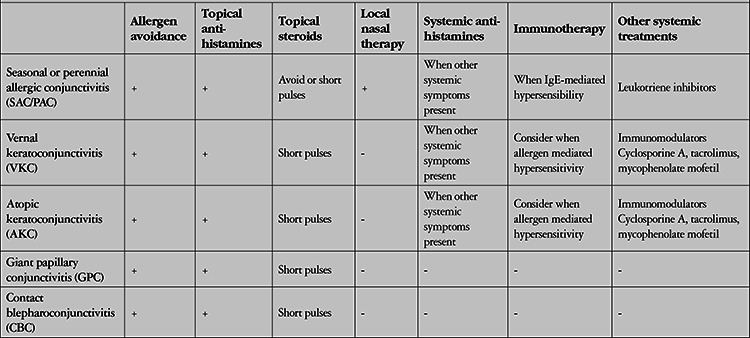
Current treatment options according to type of allergic conjunctivitis
